# A systematic review of measures of ability to meet basic needs in older persons

**DOI:** 10.1093/ageing/afad121

**Published:** 2023-10-30

**Authors:** Taryn Williams, Leon Geffen, Sebastiana Kalula, Dan J Stein, Jotheeswaran Amuthavalli Thiyagarajan, Christopher Mikton, Theresa Diaz

**Affiliations:** The Samson Institute for Ageing Research, Cape Town, South Africa; SAMRC Unit on Risk & Resilience for Mental Disorders, Department of Psychiatry and Neuroscience Institute, University of Cape Town, Cape Town, South Africa; The Samson Institute for Ageing Research, Cape Town, South Africa; The Albertina and Walter Sisulu Institute of Ageing in Africa, Department of Medicine, University of Cape Town/Groote Schuur Hospital, International Longevity Centre, Cape Town, South Africa; The Albertina and Walter Sisulu Institute of Ageing in Africa, Department of Medicine, University of Cape Town/Groote Schuur Hospital, International Longevity Centre, Cape Town, South Africa; SAMRC Unit on Risk & Resilience for Mental Disorders, Department of Psychiatry and Neuroscience Institute, University of Cape Town, Cape Town, South Africa; Ageing and Health Unit, Department of Maternal, New-born, Child, Adolescent Health and Ageing, World Health Organization, Geneva, Switzerland; Demographic Change and Healthy Ageing Unit, Department of Social Determinants of Health, World Health Organization, Geneva, Switzerland; Epidemiology, Monitoring and Evaluation Unit, Department of Maternal, Newborn, Child, Adolescent Health and Ageing, World Health Organization, Geneva, Switzerland

**Keywords:** ability to meet basic needs, older people, measures to meet basic needs, systematic review

## Abstract

**Background:**

The ability of older persons to meet their basic needs (i.e. personal, financial and housing security), as well as to perform Activities of Daily Living (ADL), is crucial. It is unclear, however, whether such measures exist. This systematic review aimed to review English-language measures of the ability of older persons to meet their basic needs, and to critically review the comprehensiveness of these measures and their psychometric properties.

**Methods:**

Fifteen electronic databases including PubMed, EBSCOhost and CINAHL were systematically searched for studies of measures that assessed the ability of older persons to meet their basic needs, as defined by the World Health Organization. Two review authors independently assessed the studies for inclusion in the review and evaluated their comprehensiveness and psychometrics.

**Results:**

We found seven instruments from 62 studies that assessed multi-domain function including ADL and some elements of basic needs. The instruments varied in breadth and in reporting of key psychometric criteria. Further, no single instrument provided a comprehensive assessment of the ability of older persons to meet their basic needs.

**Conclusion:**

No single instrument that measures the ability to meet basic needs was identified by this review. Further research is needed to develop an instrument that assesses the ability of older persons to meet their basic needs. This measure should include an evaluation of ADL.

## Key Points

- No single instrument that measures ability to meet basic needs was identified.
- Further research is needed to develop an instrument that assesses the ability of older persons to meet their basic needs.
- Measures need to include activities of daily living (ADL) performance and capacity.

## Introduction

The ability to meet basic needs is one of most fundamental requirements for older people; it serves as a foundation for ensuring an adequate standard of living [1, 2]. This ability includes older people being able to afford an adequate diet, clothing, suitable housing, and healthcare and long-term care services. It also extends to having support to minimize the impact of economic shocks that may come with illness, disability, losing a spouse or the means of livelihood [1]. The ability to meet basic needs is based on an individual’s intrinsic capacity as well as the environment in which they live, and the interaction between the two [1].

It is notable that the World Health Organization 2015 definition of the ability to meet basic needs of older persons includes personal security, and it also takes cognizance of the ability to perform basic Activities of Daily Living (ADL), such as bathing or showering, dressing, eating, getting in or out of bed or chairs, using the toilet and getting around inside the home, a topic that has previously been reviewed [1]. Despite the importance of this construct, it is unclear whether measures of the abilities to meet basic needs in older people are available.

We therefore aimed to systematically review instruments of the ability to meet basic needs in older persons. We included in our review instruments of ADL. We also aimed to critically review the comprehensiveness of the instruments and their psychometric properties.

## Method

This systematic review follows the Preferred Reporting Items for Systematic Reviews [3] methodology for conducting a systematic review. We also have submitted registration of the protocol of the review prior to the literature search in the PROSPERO (ID: CRD42022300183).

### Criteria for considering studies for this review

English language studies published between 2000 and 2022, that assessed the psychometric properties (i.e. content validity, internal consistency, criterion validity, construct validity, reproducibility, responsiveness, floor and ceiling effects and interpretability) of instruments of older persons (aged 60 years or over) ability to meet their basic needs were considered for inclusion in this systematic review.

We expected that instruments of ability to meet basic needs in older persons might be done in those with different levels of function over the course of ageing, as well as in different settings (community and long-term care facilities, healthcare facilities, old age homes, nursing homes, residential care facilities, congregate settings and frail care settings). We also expected that some instruments would assess ADL.

Instruments considered for inclusion were based on types of measures classified as psychometric measures, for example, surveys, self-report measures, questionnaires, tools, scales, checklists, test batteries, interview schedules and multi-dimensional instruments. The domain and main outcomes considered for assessment were the ability to meet basic needs, which includes personal security, financial security and adequate housing [4].

### Search methods for identification of studies

Initially we conducted our search using the following databases and registries: PubMed (includes MEDLINE), EBSCOhost (which includes Academic Search Premier and PsyINFO), CINAHL (via EBSCOhost), WHO Global Index Medicus, Global Gateway for Ageing and Maelstrom. However, given the scarcity of studies found, we extended our search and further reviewed the following additional sites: Sociological Abstracts, ERIC, AgeLine, Social Work Abstracts, International Bibliography of the Social Sciences, Social Services Abstracts, ProQuest Criminal Justice, ASSIA and SAGE.

The following search terms formed the basis of the review (also see Appendix 1 for the detailed search strategy and MeSH terms):

‘older persons’ OR ‘ageing’ OR ‘healthy ageing’ AND ‘functional ability’ OR ‘functional capacity’ OR ‘functional disability’ OR ‘basic needs’ OR ‘basic activities’ OR, ‘personal security’ OR ‘financial security’ OR ‘housing security’ AND ‘measures’ OR ‘functional assessment’ OR ‘measures of independent living’ OR ‘measures of dependency’ OR ‘disability instruments’ AND ‘community and long care facilities’ OR ‘healthcare’.

### Data collection and analysis

All studies were imported into EndNote 20 for screening. Thereafter, the automatic function and manual check was used to identify possible duplicates. Once the reviewers identified and removed duplicate records, two independent reviewers assessed the studies for inclusion and exclusion. First, the title and abstracts of each study were screened for study eligibility. Subsequently, the full-text articles were retrieved by each reviewer and independently verified for eligibility. Disagreements were resolved through consensus, or with a third reviewer.

Types of information considered for extraction centred around the description of the studies (including the authors names, title of study, year of publication, funding and type of study), characteristics of participants, measures and types of measures, measurement properties (based on the COSMIN Checklist published by [5] and 10 criteria) and outcomes.

A narrative synthesis was used to characterize the studies included in the systematic review, along with a comparative table summarising our main findings.

**Table 1:** Characteristics of measures with relevant domains

No	Instrument	Authors	Setting	Population	Language/versions	Scale domains/items
1	Katz Index of Independence in Activities of Daily Living (ADL)	Katz et al. (1959) (see [6]) Review: Pashmdarfard and Azad (2020) [6]	Community and care settings	Older adults	English, Brazilian, Turkish, Swedish, Persian	BADLs: bathing, dressing, transference, toileting, feeding and continence
2	Barthel Index	Mahoney and Barthel (1965) [7] Reviews: Pashmdarfard and Azad (2020) [6], Hopman-Rock, van Hirtum, de Vreede and Freiberger (2019) [8] Other studies: Galeoto et al. (2019) [9], Zhang et al. (2022) [10], dos Reis et al. (2022) [11]	Rehabilitation settings, patient populations	Healthy older adults and frail older adults	English, Portuguese, British, Dutch, German, Taiwanese, Turkish, Chinese (Hong Kong), Persian	BADLs: bowels, bladder spirituality, grooming, feeding, toilet use, transfer, mobility, dressing, stairs and bathing
3	Lawton Instrumental Activities of Daily Living Scale (LIADL).	Lawton and Brody (1969) [12] Review: Pashmdarfard and Azad (2020) [6] Other Studies: Graf (2008) [13]	Hospital, nursing or rehabilitation facilities	Independent and dependent older adults	English, Australian, Spanish, Malay and Korean	IADL performance: eight activities: the ability to use a phone, shopping, meal preparation, housekeeping, laundry, the model of transportation, the responsibility for one’s medication, and the ability to handle finance
4	World Health Organization Quality of Life Questionnaire (WHOQOL)	World Health Organization (1998) [14]	Culturally diverse centres	Patients and healthy older persons	Available in 77 versions	Relevant domains: Domain III Level of independence: Activities of Daily Living, dependence on medication or treatments, work capacity; Domain IV Social relationships: personal relationships, social support; Domain V Environment: physical safety and security, home environment, work satisfaction, financial resources, health and social care: accessibility and quality, opportunities for acquiring new information and skills, participation in and opportunities for recreation/leisure activities, physical environment (pollution/noise/traffic/climate), transport
5	OARS Multidimensional Functional Assessment Questionnaire (OMFAQ)	Fillenbaum (1988) (see [8]) Review: Hopman-Rock, van Hirtum, de Vreede and Freiberger (2019) [8] Other studies: Falahati et al. (2018) [15]	Service providers, community, clinic and long-term care settings	Frail older adults	English, Afrikaans, Chinese, Dutch, French, German (for use in Austria), Greek, Persian, Brazilian, Italian, etc.	ADLs: the ability to use a phone, shopping for groceries or clothes, meal preparation, housework, transportation, medication adherence, and the ability to handle money; ADL: bathing or showering, caring for one’s own appearance, feeding, walking, getting in and out of bed, trouble getting to the bathroom on time
6	The interRAI Check-Up Self-Report	Geffen, Kelly, Hogeveen and Hirdes (2020) [16]	Community and aged residential care, nursing homes, care settings, primary health settings	Older persons	English	Losses of intrinsic capacity: ability to complete certain tasks (e.g. climb stairs), ability to carry out IADL and ADLs: continence, trade-offs, living arrangements, well-being, nutrition, and social relationships
7	Functional Independence and Difficulty Scale (FIDS)	Review: Hopman-Rock, van Hirtum, de Vreede and Freiberger (2019) [8] Other studies: Saito, Matsui and Watanabe (2017) [17], Sertel, Yümin and Özel (2022) [18]	Long-care settings	Older adults, community-dwelling older persons	English, Japanese, Turkish, etc.	Assesses both independence and subjective difficulty of BADL performance

**Table 2:** Psychometric properties and ratings of instruments

Measurement property	Rating	Personal Security/Activities of Daily Living		Financial Security		Adequate Housing/Standard of Living		
		Katz Index of Independence in Activities of Daily Living	Barthel Index	Lawton Instrumental Activities of Daily Living Scale	World Health Organization Quality of Life Questionnaire	OARS Multidimensional Functional Assessment Questionnaire	The InterRAI Check-Up Self-Report	Functional Independence and Difficulty Scale
Construct validity	Overall rating	±						
	QoE	Low						
Criterion validity	Overall rating	±	±			+		
	QoE	Low	Low			Mod		
Concurrent validity	Overall rating		±					
	QoE		Low					
Convergent validity	Overall rating	±		+			+	
	QoE	Low		Mod			Mod	
Discriminant validity	Overall rating				±			
	QoE				Low			
Internal consistency	Overall rating	±	+		+	+	+	
	QoE	Low	High		Mod	Mod	High	
Inter-rater reliability	Overall rating	±	+	+	+			±
	QoE	Low	Mod	Mod	Mod			Low
Intra-rater reliability	Overall rating		+					
	QoE		Mod					
Test re-test reliability	Overall rating		±	+	+	+		
	QoE		Low	Mod	Mod	Mod		
Responsiveness	Overall rating	+						±
	QoE	Mod						Low
Validity	Overall rating							+
	QoE							Mod

## Results

### Search results

A total of 5,832 studies were found across the identified databases and registries (see PRISMA flow diagram). Of these studies, 398 studies were duplicate records and were removed. We then screened the remaining 5,434 studies and excluded 4,958 studies, given that they did not meet the eligibility criteria of our systematic review. Four hundred and seventy-six studies were then reviewed. A further 414 studies were excluded, leaving 62 articles for consideration and inclusion. We found seven instruments in these 62 studies that assessed ADL. However, we found no measures of ability to meet basic needs.


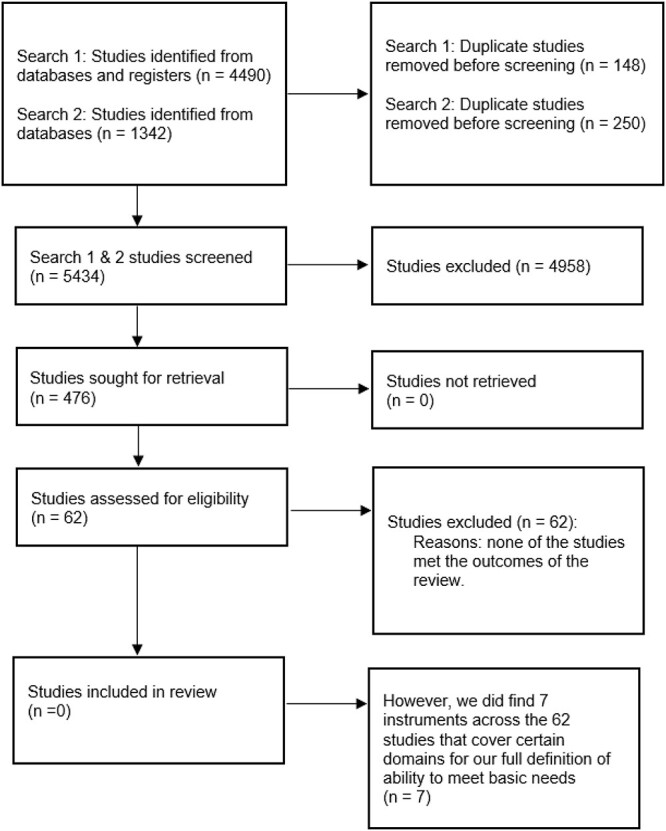


PRISMA flow diagram: identification of databases, registries and instruments.

### Results of possible measures and their psychometric properties for consideration

The seven instruments of ADL found were the Katz Index of Independence in ADL, the Barthel Index, the Lawton Instrumental Activities of Daily Living (LIADL) Scale, the World Health Organization Quality of Life Questionnaire (WHOQOL), the OARS Multidimensional Functional Assessment Questionnaire (OMFAQ), The InterRAI Check-Up Self-Report, and the Functional Independence and Difficulty Scale (FIDS). Across the seven instruments, study populations had different levels of function (ranging from independent to frail) and settings varied (ranging from community and care settings to hospital settings). These measures are available in English, and several have been translated into other languages. The seven instruments focus on ADLs and IADLs as well as on the goals of care, advance care preferences, nutrition and or weight change, living situation, standard of living, social relationships and the environment (Table 1).

Instruments also vary in strength and the reporting of key psychometric criteria, namely, construct, convergent, discriminant and criterion validity, as well as internal consistency, responsiveness, inter-rater and intra-rater reliability, and test re-test reliability. Further, no instruments were found that reported psychometric criteria on the abilities to afford an adequate diet, clothing, suitable housing, healthcare and long-term care services (Table 2).

## Discussion

Our search yielded seven instruments from 62 studies. These measures assessed multi-domain function including ADL and some elements of basic needs, and so assess some aspects of the ability to meet basic needs of older persons. The instruments varied in length and in reporting of key psychometric properties. Importantly, no comprehensive measure of the ability to meet the basic needs of older persons, as defined by the World Health Organization, was found. It is notable that the notion of ability to meet the basic needs of older persons was introduced relatively recently by the World Health Organization.

Findings from this review support a number of recommendations. First, in order to research the ability to meet the basic needs of older persons, a new measure may be useful. Second, given that instrument development is a time-consuming and a multi-year process, in the interim, measures found here, such as the InterRAI CheckUp Self Report and the WHOQOL may be useful insofar as they assess some aspects of the ability to meet basic needs of older persons [14, 16]. In addition, the Urban Resident Psychological Security Scale [19], which measures psychological security, may help determine how older persons think and feel about their personal security. Similarly, a financial needs indicator has been developed to measure financial needs in low- and middle-income countries, although it has not yet been tested in older persons may help determine financial security [20].

Several limitations of this work deserve emphasis. First, the search was confined to articles published in the English language. Second, had we searched specifically for instruments of quality of life in the older person, we might have found a range of additional measures that provide some indications of the ability to meet basic needs in this population.

In conclusion, further research is needed to develop a measure that assesses the ability of older persons to meet their basic needs. This measure should include an evaluation of ADL. In the interim, measures of ADLs or QoL, such as the InterRAI CheckUp Self Report instrument and the WHOQOL may be useful in assessing some aspects of the ability of older persons to meet their basic needs.

## Supplementary Material

aa-23-0356-File002_afad121
