# Supplementary material for: A systematic review of measures of ability to meet basic needs in older persons
Source: Age Ageing. 2023 Oct 30;52(Suppl 4):iv112–7. doi: 10.1093/ageing/afad121 (PMC10615038; doi:10.1093/ageing/afad121)
Supplement: aa-23-0356-File002_afad121 [file aa-23-0356-file002_afad121.docx]

World Health Organization: *Measurement of Healthy Ageing.*

**A systematic review of measures** **of ability to meet basic needs in older persons**

SUPPLEMENTARY DATA

**Appendix 1:** Search terms

| **Search** | **Terms** |
| --- | --- |
| #1 | (older[All Fields] AND ("persons"[MeSH Terms] OR "persons"[All Fields])) OR (60[All Fields] AND years[All Fields] AND older[All Fields]) OR ("aged"[MeSH Terms] OR "aged"[All Fields] OR ("senior"[All Fields] AND "citizen"[All Fields]) OR "senior citizen"[All Fields]) OR ("aged"[MeSH Terms] OR "aged"[All Fields]) OR ("frail elderly"[MeSH Terms] OR ("frail"[All Fields] AND "elderly"[All Fields]) OR "frail elderly"[All Fields] OR ("elderly"[All Fields] AND "frail"[All Fields]) OR "elderly, frail"[All Fields]) OR ("frail elderly"[MeSH Terms] OR ("frail"[All Fields] AND "elderly"[All Fields]) OR "frail elderly"[All Fields] OR ("frail"[All Fields] AND "elders"[All Fields]) OR "frail elders"[All Fields]) OR "elder"[All Fields] OR seniors[All Fields] OR ("aging"[MeSH Terms] OR "aging"[All Fields] OR "ageing"[All Fields]) OR ("aged"[MeSH Terms] OR "aged"[All Fields] OR ("older"[All Fields] AND "adults"[All Fields]) OR "older adults"[All Fields]) OR old[All Fields] OR "independent"[All Fields] OR ("dependent"[All Fields] AND "living"[All Fields]) |
| #2 | (functional[All Fields] AND capacity[All Fields]) OR ("activities of daily living"[MeSH Terms] OR ("activities"[All Fields] AND "daily"[All Fields] AND "living"[All Fields]) OR "activities of daily living"[All Fields] OR ("functional"[All Fields] AND "ability"[All Fields]) OR "functional ability"[All Fields]) OR (functional[All Fields] AND disability[All Fields]) OR (intrinsic[All Fields] AND capacity[All Fields]) OR (basic[All Fields] AND ("health services needs and demand"[MeSH Terms] OR ("health"[All Fields] AND "services"[All Fields] AND "needs"[All Fields] AND "demand"[All Fields]) OR "health services needs and demand"[All Fields] OR "needs"[All Fields])) OR (dependent[All Fields] AND living[All Fields]) OR ("independent living"[MeSH Terms] OR ("independent"[All Fields] AND "living"[All Fields]) OR "independent living"[All Fields]) OR personal[All Fields] AND security[All Fields] OR ("economics"[MeSH Terms] OR "economics"[All Fields] OR "financial"[All Fields]) AND security[All Fields] OR "socioeconomic factors"[MeSH Terms] OR ("socioeconomic"[All Fields] AND "factors"[All Fields]) OR "socioeconomic factors"[All Fields] OR ("standard"[All Fields] AND "living"[All Fields]) OR "standard of living"[All Fields] |
| #3 | ("weights and measures"[MeSH Terms] OR ("weights"[All Fields] AND "measures"[All Fields]) OR "weights and measures"[All Fields] OR "measures"[All Fields]) OR (("psychometrics"[MeSH Terms] OR "psychometrics"[All Fields] OR "psychometric"[All Fields]) AND ("weights and measures"[MeSH Terms] OR ("weights"[All Fields] AND "measures"[All Fields]) OR "weights and measures"[All Fields] OR "measures"[All Fields])) OR ("weights and measures"[MeSH Terms] OR ("weights"[All Fields] AND "measures"[All Fields]) OR "weights and measures"[All Fields] OR "scales"[All Fields]) OR tools[All Fields] OR ("surveys and questionnaires"[MeSH Terms] OR ("surveys"[All Fields] AND "questionnaires"[All Fields]) OR "surveys and questionnaires"[All Fields] OR "questionnaires"[All Fields]) OR ("checklist"[MeSH Terms] OR "checklist"[All Fields] OR "checklists"[All Fields]) OR (("self-report"[MeSH Terms] OR ("self"[All Fields] AND "report"[All Fields]) OR "self-report"[All Fields]) AND ("weights and measures"[MeSH Terms] OR ("weights"[All Fields] AND "measures"[All Fields]) OR "weights and measures"[All Fields] OR "measures"[All Fields])) OR (“functional” [All Fields] AND "assessment" [All Fields]) OR "measures"[All Fields]) AND ("independent living"[MeSH Terms] OR ("independent"[All Fields] AND "living"[All Fields]) OR "independent living"[All Fields])) OR "measures"[All Fields]) AND ("dependency, psychological"[MeSH Terms] OR ("dependency"[All Fields] AND "psychological"[All Fields]) OR "psychological dependency"[All Fields] OR "dependency"[All Fields])) |
| #4 | ("residence characteristics"[MeSH Terms] OR ("residence"[All Fields] AND "characteristics"[All Fields]) OR "residence characteristics"[All Fields] OR "community"[All Fields]) AND (long[All Fields] AND care[All Fields] AND facilities[All Fields]) OR (residential[All Fields] AND care[All Fields] AND facilities[All Fields]) OR ("nursing homes"[MeSH Terms] OR ("nursing"[All Fields] AND "homes"[All Fields]) OR "nursing homes"[All Fields]) OR ("homes for the aged"[MeSH Terms] OR ("homes"[All Fields] AND "aged"[All Fields]) OR "homes for the aged"[All Fields] OR ("old"[All Fields] AND "age"[All Fields] AND "homes"[All Fields]) OR "old age homes"[All Fields]) OR (older[All Fields] AND ("persons"[MeSH Terms] OR "persons"[All Fields] OR "person"[All Fields]) AND congregate[All Fields] AND settings[All Fields]) OR (frail[All Fields] AND care[All Fields]) OR (assisted[All Fields] AND living[All Fields]) OR "delivery of health care"[MeSH Terms] OR ("delivery"[All Fields] AND "health"[All Fields] AND "care"[All Fields]) OR "delivery of health care"[All Fields] OR "healthcare"[All Fields] |
